# The Mitogen-Activated Protein Kinase p38α Regulates Tubular Damage in Murine Anti-Glomerular Basement Membrane Nephritis

**DOI:** 10.1371/journal.pone.0056316

**Published:** 2013-02-18

**Authors:** Ralf Müller, Christoph Daniel, Christian Hugo, Kerstin Amann, Dirk Mielenz, Karlhans Endlich, Tobias Braun, Betty van der Veen, Peter Heeringa, Georg Schett, Jochen Zwerina

**Affiliations:** 1 Department of Internal Medicine 3, University of Erlangen-Nuremberg, Erlangen, Bavaria, Germany; 2 Department of Internal Medicine 4, University of Erlangen-Nuremberg, Erlangen, Bavaria, Germany; 3 Medical Clinic III, Technical University of Dresden, Dresden, Saxony, Germany; 4 Department of Pathology, University of Erlangen-Nuremberg, Erlangen, Bavaria, Germany; 5 Institute of Anatomy and Cell Biology, University of Greifswald, Mecklenburg-West Pomerania, Germany; 6 Department of Pathology and Medical Biology, University Medical Center Groningen, University of Groningen, Groningen, The Netherlands; University of Illinois at Chicago, United States of America

## Abstract

p38 mitogen-activated protein kinase (MAPK) is thought to play a central role in acute and chronic inflammatory responses. Whether p38MAPK plays a pathogenic role in crescentic GN (GN) and which of its four isoforms is preferentially involved in kidney inflammation is not definitely known. We thus examined expression and activation of p38MAPK isoforms during anti-glomerular basement membrane (GBM) nephritis. Therefore, p38α conditional knockout mice (MxCre-p38α^Δ/Δ^) were used to examine the role of p38α in anti-GBM induced nephritis. Both wild type and MxCre-p38α^Δ/Δ^ mice developed acute renal failure over time. Histological examinations revealed a reduced monocyte influx and less tubular damage in MxCre-p38α^Δ/Δ^ mice, whereas glomerular crescent formation and renal fibrosis was similar. Likewise, the levels of pro- and anti-inflammatory cytokines such as TNF, IL-1 and IL-10 were similar, but IL-8 was even up-regulated in MxCre-p38α^Δ/Δ^ mice. In contrast, we could detect strong down-regulation of chemotactic cytokines such as CCL-2, -5 and -7, in the kidneys of MxCre-p38α^Δ/Δ^ mice. In conclusion, p38α is the primary p38MAPK isoform expressed in anti-GBM nephritis and selectively affects inflammatory cell influx and tubular damage. Full protection from nephritis is however not achieved as renal failure and structural damage still occurs.

## Introduction

The MAPK family comprises a large group of protein kinases that respond for example to growth factors, osmotic stress, ultraviolet light and cytokines to regulate cell proliferation, differentiation and apoptosis [1]–[4]. MAPK regulate three major pathways: the Jun N-terminal kinases (JNKs), the extracellular signal-related kinases (ERKs) and the p38 MAPKs [5]. The p38MAPK pathway was initially identified in macrophages stimulated with lipopolysaccharide (LPS) and is present in many cells and tissues [6], [7]. Pro-inflammatory cytokines can stimulate signal transduction through upstream kinases finally resulting in the phosphorylation and activation of p38MAPK. In turn, p38MAPK phosphorylates other kinases such as MAPKAPK2 (MK2) and activating transcription factor 2 (ATF2), which promote transcription of pro-inflammatory genes [8].

p38MAPKs are represented by four different isoenzymes: p38α, p38β, p38γ and p38δ [9]–[14]. Recently, the *in vivo* functions of the four isoenzymes could be partially defined. p38β, p38γ and p38δ are activated by distinct stimuli *in vitro* and are expressed in a more restricted manner. However, mice deficient in either one of these isoenzymes do not show a major phenotype [15], [16]. In contrast, p38α plays an important role in tissue homeostasis and is widely expressed. In fact, p38α-deficient mice are not viable due to placental defects [17]–[19]. Recently, the use of mice conditionally deficient for p38α revealed specific roles of this isoenzyme in erythropoiesis as well as cardiac and liver regeneration [20].

Besides the developmental and regenerative function of p38α, a pro-inflammatory role has been proposed based on the pharmacological inhibition of p38 in several animal models of acute and chronic inflammation. Neutralization of p38 ameliorates pro-inflammatory cytokine production and tissue damage in mouse models of arthritis and other autoimmune disease models [21]–[25]. Moreover, p38α inhibitors were successfully used in a rodent model of crescentic glomerulonephritis (GN) [26], [27]. Blockade of p38α was associated with reduction in infiltrating leukocytes and subsequent tissue damage. However, some of these previously used p38 inhibitors are not entirely specific for p38MAPK and block both the α- and β-isoform. Also, such inhibitors showed only minor and transient efficacy in a clinical trial in patients with rheumatoid arthritis [28].

Thus, it is yet unclear whether p38α indeed plays a specific role in crescentic GN and whether its inhibition could emerge as an effective treatment for this rapidly progressive autoimmune disease. In this study, we thus used mice conditionally deleted for p38α and induced anti-glomerular basement membrane nephritis (anti-GBM) to test whether p38α is indeed responsible for tissue damage and leukocyte infiltration in kidneys affected by crescentic GN.

## Materials and Methods

### Animals

*MxCre-p38α^Δ/Δ^* mice and *MxCre-p38α^fx/fx^* mice (wild type littermates, genetic background C57Bl/6) were used for the experiments [20]. The deletion of the floxed alleles was induced by injecting 13 mg/kg polyinosinic-polycytidylic acid (Sigma-Aldrich) for 3 times intraperitoneally at week 10 of age. Genotyping of mice was performed in all mice. (Primers for genotyping are given in Text S1). All animal experiments were approved by the animal ethics committee of the government of franconia (permit number 54-2532.1-11/10) and were carried out according to legal obligations defined by national animal protection laws.

### Induction of Anti-glomerular Basement Membrane (GBM) Glomerulonephritis (GN)

Accelerated anti-GBM GN was induced in *MxCre-p38α^Δ/Δ^* and wildtype mice as described previously by Asgeirsdottir *et al* [29]. Briefly, 11-week-old mice were intraperitoneally immunized with 200 µg of sheep IgG (Sigma-Aldrich) dissolved in complete Freund´s adjuvant. After 6.5 days, mice received an intravenous injection of sheep anti-mouse GBM antibodies (50 mg/kg) and 200 ng of recombinant mouse tumor necrosis factor α (TNFα) in a total volume of 200 µl. Mice were sacrificed after indicated time periods and kidneys were perfused with 0.9% cold sodium chloride solution. One kidney was fixed in 4% formaldehyde for histology while the other kidney was divided into 3 parts and snap-frozen in liquid nitrogen and stored at −80°C for protein and mRNA analysis.

### Blood and Urine Examinations

Blood urea nitrogen (BUN) was measured in serum at days 3, 7 and 14 after inducement of anti-GBM GN. A quantitative enzymatic colorimetric BUN determination kit (Stanbio Laboratory, Boerne, Texas) was used according to the manufacturer’s protocol. To determine kidney function, mice were placed in single metabolic cages for 24 h the day before sacrifice. Creatinine was measured in serum and urine, and creatinine clearance was calculated according to the following formula: crea[urine] × vol[urine]/crea[plasma].

### Murine Podocyte Cell Culture

Conditionally immortalized mouse podocyte cell lines were kindly provided by Prof. Karlhans Endlich (University of Greifswald). They were isolated from kidneys of Immorto-Mouse® mice (Charles River, St. Louis, MO) and carry a thermosensitive variant of the SV-40 large T-antigen as a transgene. T-antigen expression is under control of the H-2K^b^ promoter and can be induced by mouse interferon-γ (IFNγ). Proliferating cells are cultured under permissive conditions (IFNγ) at 33°C. Cells differentiate within 14 days when grown under non-permissive conditions (without IFNγ) at 37°C ^42^. Before thawing the cells a tissue culture flask was coated with collagen from rat tail (0.1 mg/ml; BD Biosciences, Bedford, MA) in phosphate buffered saline (PBS) for 1 h at 37°C. Cells were plated and propagated at 33°C in RPMI 1640 medium (Gibco) containing 10% FBS (PAN-Biotech, Aidenbach, Germany), 100 µg/ml penicillin-streptomycin (Gibco) and 20 U/ml γ-interferon (Sigma). For differentiation growing cells were trypsinized and replated in non-coated flasks in the medium described above but without IFNγ. Cells were used at a maximum confluence.

To assess p38 MAPK isoform activation, differentiated podocytes were stimulated with recombinant murine TNFα (10 ng/ml, Biosource) after starvation for 12 hours. Cells were stimulated with TNFα for 60, 30, 15, 10, 5 and 0.5 minutes. After the indicated time points, cells were lysed, lysates were mixed with 2× sample loading buffer (SLB; 4% SDS, 125 mM Tris/HCl pH 6.8, 10% Glycerol, 100 mM DTT, 0.002% bromphenolblue), boiled and stored at −20°C until western blot analysis.

### Primary Antibodies

The following antibodies were used: rabbit anti-phospho p38MAPK (1∶300; Cell Signaling Technology, Beverly, MA), rabbit anti-p38MAPK (1∶300; Cell Signaling), rabbit anti-p38MAPKα (1∶300; Cell Signaling), rabbit anti-p38MAPKβ (1∶300; Abgent, San Diego, CA), rabbit anti-p38MAPKγ (1∶300; Cell Signaling), rabbit anti-p38MAPKδ (1∶300; Abgent), rabbit anti-phospho MK2 (1∶300; Cell Signaling), rabbit anti-MK2 (1∶300; Santa Cruz Biotechnology, Santa Cruz, CA), rabbit anti-phospho ATF-2 (1∶300; Cell Signaling), rabbit anti-ATF-2 (1∶300; Santa Cruz), rabbit anti-phospho MKK3/6 (1∶300; Santa Cruz), rabbit anti-MKK3 (1∶300; Santa Cruz), rabbit anti-MKK6 (1∶300; Santa Cruz), rabbit anti-βActin (1∶400; Sigma-Aldrich, St. Louis, MO). Antibodies against macrophages (anti-F4/80; 1∶100; Serotec), neutrophils (1∶300; MorphoSys), T lymphocytes (anti CD3; 1∶100; NeoMarkers) and B cells (anti-CD20; 1∶100; Sigma) were used for immunohistochemistry.

### Western Blotting

*In vitro* cultured podocytes were lysed, lysates were mixed with 2× SLB, boiled and separated by SDS-PAGE followed by transfer onto nitrocellulose membrane. After blocking with 10× Tris-buffered saline (TBS), 0.1% Tween 20 and 5% non fat dry milk, membranes were incubated with primary antibodies. Appropriate secondary horseradish peroxidase-conjugated antibodies (Dako, Glostrup, Denmark) and a chemoluminescent detection system (Pierce, Rockford, IL) were applied. The phosphorylated MAPKs were analyzed by normalization to total amount of kinase. For western blotting analysis of kidneys, protein lysates from frozen tissues were prepared. Tissues were dissolved in buffer containing urea (7M), glycerol (10%), SDS (1%), Tris pH 6,8 (10 mM), phosphatase inhibitors (Sigma) and protease inhibitors (Roche, Mannheim, Germany). Each piece of tissue was homogenized with an Ultra Turrax and centrifuged for 15 min with 15.000 g at 4°C to get rid of tissue debris. The supernatant was transferred and protein concentration determined (BCA protein assay kit, Pierce). Western Blotting was performed as described above.

### Immunoprecipitation

To determine p38 MAPK isoform phosphorylation in cultured podocytes and whole kidney tissues, immunoprecipitation was performed. For precipitation of cells, differentiated growth arrested podocytes were used after 15 min stimulation with TNFα (10 ng/ml). Cells were lysed in buffer (NP40 1%, sodium chloride 150 mM, Tris/HCl pH 7,5 25 mM, EDTA 1 mM, EGTA 1 mM, sodium fluoride 1 mM, ß-glycerophosphat 1 mM, sodium pyrophosphate 2,5 mM, vanadate 1 mM, PMSF 1 mM) for 20 minutes on ice, followed by 10 min centrifugation at 10.000 g at 4°C. After determination of protein concentration, samples were mixed with 30 µl of immobilized protein A plus sepharose (Pierce) and antibody against phospho-p38 MAPK (1∶50) and incubated at 4°C for 2 h while gently shaking. After 3 washing steps with lysis buffer (5 min, 1.000 g, 4°C) the pellet was resuspended, boiled in 1× SLB and stored at −20°C. As controls two further immunoprecipitations were performed: One without lysate (negative control) and one with an isotype matched control antibody for phospho-p38 MAPK. Immunoprecipitation of kidney tissue taken at day 3 after injection of nephrotoxic serum was done by homogenizing the frozen tissue with an Ultra Turrax in buffer at 4°C as described above with addition of sodium dodecylsulfate (0.1%). After centrifugation (20 min, 10,000 g, 4°C) the supernatant was taken and the same procedure followed as described above. For western blot analysis each gel pocket was loaded with the full IP preparation or 50 µg of cell or tissue lysate.

### Quantitative Real-time RT-PCR

RNA was isolated from cells and tissue with peqGold TriFast reagent (Peqlab, Erlangen, Germany). RNA was isolated following standard laboratory procedures with chloroform and alcoholic precipitation. Purity was measured by photometery (Eppendorf). 1 µg of RNA was reversely transcribed using MuLV reverse transcriptase (Darmstadt, Germany) and random hexamer primers. Quantitative PCR amplifications were performed according to manufacturer’s protocol on an ABI Prism 7300 sequence detection system (Applied Biosystems). Primer sequences are given in Text S1. The RT^2^ Profiler™ PCR Array System (SABiosciences, Frederick, MD, USA) for mouse chemokines and receptors was used to analyze the expression of a focused panel of genes. To this end we isolated RNA from five wild type and five *MxCre-p38α^Δ/Δ^* mice 14 days after induction of anti-GBM induced nephritis. RNA isolation, cDNA preparation and PCR were done according to the manufactureŕs protocol. Thermal cycling was run in an ABI Prism 7300 sequence detection system (Applied Biosystems).

### Histology

To determine the extent of crescent formation, tubular dilatation and scarring, paraffin embedded sections were stained with Sirius red, periodic acid-Schiff reagent (PAS) and hematoxylin and eosin (HE). To assess the percentage of crescents, all glomeruli of a section were counted. Afterwards the ratio of glomeruli affected by crescents to unaffected glomeruli was determined. The tubular damage index (0 to 3) was determined by assessing inflammation, fibrosis, tubular dilatation and tubular atrophy. Fibrosis scoring was performed semiquantitatively on Sirius red stained sections by evaluating the extent of fibrotic tissue vs. normal kidney tissue. All analyses were done with an Olympus CX41 microscopy and a fence ocular in 200× magnification.

### Immunohistochemistry

To determine immune cell infiltration of kidneys, serial sections (2 µm) of paraffin-embedded murine kidneys were used for immunohistochemistry. Slides for detection of T cells, neutrophils and macrophages were pretreated with recombinant protease K (Roche, Mannheim), whereas B-cell detection required no pretreatment. Non-specific binding was blocked by addition of a mixture (1∶1 vol) of 10% goat serum and Roti-Immunoblock (Carl Roth, Karlsruhe, Germany). Sections were incubated with primary antibodies for 1 h at room temperature, followed by 30 min incubation with specific biotinylated immunoglobulin (Vector, Burlingame, CA). Vectastain ABC reagent (Vector) and 3,3′-diaminobenzidine (DAB, Sigma-Aldrich) as chromogen were used for final detection. Sections were counterstained with haematoxylin was done. Scoring of infiltrated immune cells was as follows: 20 lens coverages were counted all over the section (mark and cortex) and indicated in positive cells/mm^2^.

### Statistical Analysis

Results were analyzed for statistical differences either with one-way ANOVA and Tukeýs multiple comparison test or unpaired t-test. P≤0.05 was considered statistically significant. Data are presented as the mean±SEM.

## Results

### Selective Activation of p38α during Anti-GBM Nephritis

To determine whether p38MAPK signalling is active during crescentic GN, we used an established anti-GBM model and evaluated gene and protein expression during the disease course. To this end, we immunized mice with sheep IgG and then challenged the sensitized mice with sheep anti-GBM antibodies in conjunction with a single TNFα injection as previously described [29].

**Figure 1:**
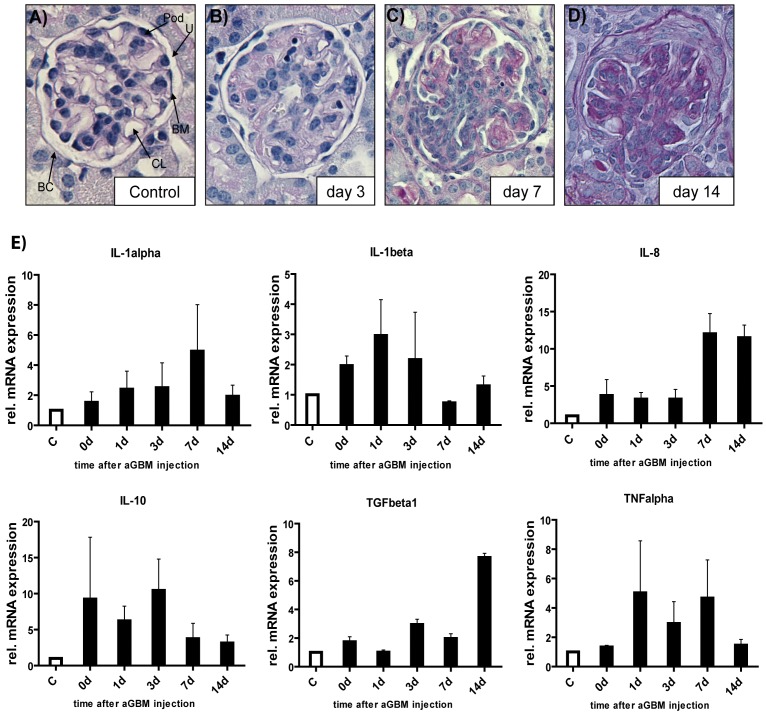
Functionality of the anti- GBM nephritis model and differential cytokine activation. Periodic acid-Schiff reagent (PAS) stain of a control kidney (A) and kidneys affected by crescentic GN over time are shown. BC = Bowmańs capsule, CL = capillary loop, BM = basement membrane, Pod = podocyte, U = urinary space. Development of full crescents occurs within 14 days (B–D). Mice were sacrificed at indicated days (original magnification 20×). (E) Expression analyses of pro- (TNFα, IL-1, IL-8) and anti-inflammatory (IL-10, TGFβ1) cytokines by qPCR in kidneys of control mice (white bars) and anti GBM-IgG treated mice (black bars). RNA was isolated from whole kidney lysates. Data are the mean value ± SEM (n = 4 for each time point).

To demonstrate the functionality of our model, we performed histological analysis during different time points. As shown in Fig. 1A–D, injection of anti-GBM antibodies causes glomerular inflammation, glomerular crescent formation and tubular damage during the course of disease. We then analyzed expression of pro-inflammatory (TNFα, IL-1, IL-8) and anti-inflammatory (IL-10, TGFβ1) cytokines by quantitative real-time PCR (qPCR) to gain insight into the molecular changes during the inflammatory response (Fig. 1E). TNFα and IL-1β mRNA were up regulated during the first 7 days of disease. In contrast, IL-8 was found to be over-expressed during later stages of disease. We also found early up-regulation of the counter-regulatory cytokine IL-10 during anti-GBM induced nephritis. Interestingly, TGFβ1 was over-expressed at late stages of anti-GBM nephritis, which might correlate to crescent formation occurring at the same time.

We next analyzed the expression and activation of the p38MAPK pathway in this model. We therefore investigated early (35 min) and late (14 days) time points after injection of anti-GBM antibodies. First, we determined expression of the four known p38MAPK isoforms during the course of disease and could detect mRNA expression of all four p38MAPK isoforms in kidney lysates. Expression of all four isoforms was however not different during the disease course (*data not shown*). We then performed Western Blot analysis to confirm these findings. In support of the mRNA data, we could detect p38α, p38β, p38γ and p38δ in kidneys injected with anti-GBM antibodies. In line with results from qPCR, protein expression remained stable throughout the observation period (*data not shown*).

**Figure 2:**
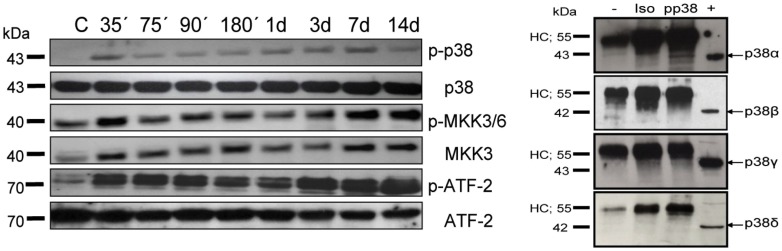
Selective activation of p38α during anti- GBM nephritis. (**A**) p38MAPK phosphorylation during anti-GBM induced nephritis was investigated at early (35 min) and late (14 days) time points after disease induction using phospho-specific antibodies (left graph). Control lane shows no p38 phosphorylation before injection of anti- GBM antibodies. (**B**) Co-Immunoprecipitation (IP) was used to determine which p38 isoform is activated during anti- GBM nephritis. Kidney tissue was retrieved three days after induction of glomerulonephritis. IP was performed with buffer only (−) or with kidney lysates and anti-phospho p38MAPK antibody, or with kidney lysates using an isotype-matched control antibody (Iso). IPs and positive control lysate (+) were separated by SDS-PAGE, blotted onto nitrocellulose and probed with specific antibodies against p38 isoforms (arrows). HC: heavy chain of the precipitating antibody (right graph).

After assuring the expression of p38MAPK isoforms in anti-GBM induced nephritis, we asked whether the p38MAPK pathway is active during disease. As shown in Fig. 2, p38MAPK is phosphorylated and thus active throughout the observation period of anti-GBM induced nephritis. In addition, we found that the upstream kinase MKK3/6 and the downstream transcription factor ATF-2 is also phosphorylated indicating a functional p38MAPK signalling pathway in inflamed kidneys. To investigate, which p38MAPK isoform is preferentially activated during anti-GBM induced nephritis we analyzed the activation status of the p38MAPK isoforms using an activation-specific anti-phospho p38MAPK antibody. Interestingly, we found a clear activation of the α-isoform, but not the other p38MAPK isoforms (Fig. 2). Thus, p38MAPK is expressed and the only active p38MAPK isoform during anti-GBM induced nephritis is the α-isoform of p38.

### Pro-inflammatory Cytokine Stimulation Specifically Activates p38α in Podocytes *in vitro*

We next investigated whether the p38MAPK pathway is functional in podocytes *in vitro* as these cells are critically involved in experimental nephritis and crescent formation [30], [31]. As described previously, we therefore cultured undifferentiated and differentiated immortalized podocytes *in vitro* and stimulated them with TNFα [32]. Analysis of mRNA abundance of the four p38 MAPK isoforms and up- and downstream kinases revealed expression of the p38 α and γ isoform as well as of MKK3, MKK6, MK2 and ATF-2 (Fig. S1A–B). These findings were supported by western blot analyses for the four isoforms (Fig. S1C): p38MAPK α and γ but not β and γ were detectable especially in differentiated podocytes. Next, we stimulated differentiated podocytes with TNF-α (10 ng/ml) to mimic an inflammatory condition. Western blot analyzes revealed enhanced signalling as early as 5 minutes (p-p38) to 10 minutes (p-MK2, p-ATF-2) after stimulation (Fig. S1D) suggesting that podocytes turn on p38MAPK signalling within minutes after cytokine exposure. We then addressed which of the p38MAPK isoform is activated. Immunoprecipitation using an activation-specific anti-phospho p38MAPK antibody (Fig. S1E) showed very clearly that only p38α is detectable and thus active. Thus, we conclude that pro-inflammatory stimuli preferentially activate the α-isoform of p38MAPK further supporting the use of *MxCre-p38α* ^Δ/Δ^ mice in the anti- GBM induced nephritis model.

### Effects of Conditional p38α Deletion on Anti-GBM Nephritis

**Figure 3:**
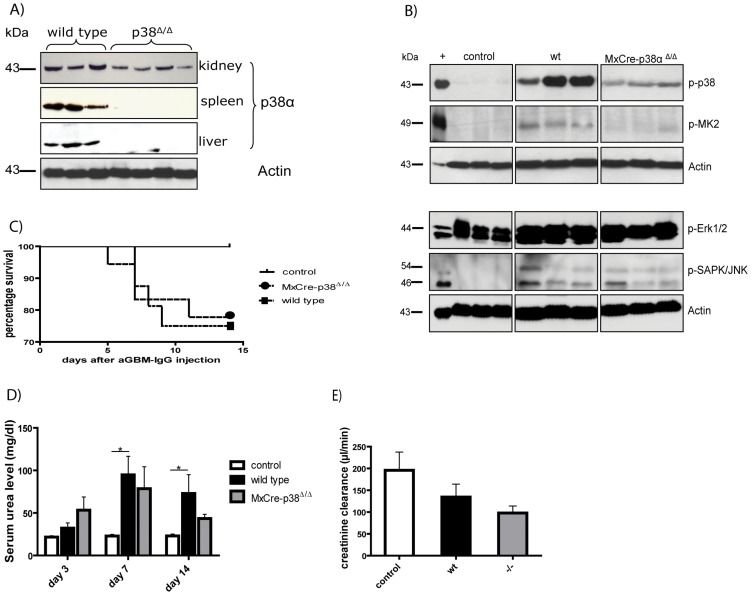
Effects of conditional p38α deletion on anti-GBM nephritis. (A) p38MAPKα deletion is depending on tissue and reaches from 50–100%. (B) Western blot experiments reveal significant reduction of p38 phosphorylation in kidneys of *MxCre-p38α^Δ/Δ^* transgenic mice at day 14 after induction of anti-GBM nephritis. Downstream activation of MK2 is also strongly reduced. Three representative animals are shown in each group. (C) Survival of wild type and *MxCre-p38α^Δ/Δ^* mice during anti-GBM nephritis shows no difference (controls n = 10, continuous line; wild type n = 16, spotted line; *MxCre-p38α^Δ/Δ^* mice n = 18, dashed line). (D) Serum urea levels in wild-type (day 14: mean 66.61±18.33 mg/dl; n = 9) and *MxCre-p38α^Δ/Δ^* mice (day 14: mean 58.43±10.46 mg/dl, p = ns vs. wild-type; n = 9). (E) Creatinine clearance decreases in both wild type (134.4±29.70; n = 8) and *MxCre-p38α^Δ/Δ^* (97.79±16.15; n = 8) mice.

First, we determined whether p38-gene deletion is evident in the *p38α* ^Δ/Δ^ transgenic mice [20]. As represented in Fig. 3A there is a full knockout in tissue like spleen and liver and an approximately 50% deletion in the kidney. This is due to the *MxCre-flox* model and confirmed by other studies. Nevertheless this model is suitable for the investigation of anti-GBM nephritis, because the deletion occurs in areas, which are of interest in this study, e.g. vascular endothelium, glomerulus, distal convoluted tubule and collecting duct [33], [34]. We found significant reduction of p38 phosphorylation in kidney lysates. In support of these data, downstream activation of MK2 was also strongly reduced. Indeed other MAP kinases like ERK and JNK are also activated in this model, but there is no further regulation detectable in the case of *p38α^Δ/Δ^* (Fig. 3B). After p38α deletion, we induced anti-GBM nephritis as described above. We then determined survival of wild type and *MxCre-p38α^Δ/Δ^* mice during the course of nephritis. Unexpectedly, we found no difference in the survival curve as shown in Fig. 3C. Also, serum urea levels increased both in wildtype (day 14: mean 66.6±18.3 mg/dl) and *MxCre-p38α^Δ/Δ^* mice (day 14: mean 58.4±10.5 mg/dl, p = ns vs. wild type) similarly compared to healthy mice (Fig. 3D). Creatinine clearance revealed a decrease in both wildtype (134.4±29.70; n = 8) and *MxCre-p38α^Δ/Δ^* (97.79±16.15; n = 8) mice (Fig. 3E) in contrast to healthy control mice (195.8±41.88; n = 4) suggesting impaired filtration function of the kidney. Thus, deletion of p38α does not protect from kidney failure in this nephritis model.

### Deletion of p38α Ameliorates Tubular but not Glomerular Damage during Anti- GBM Nephritis

**Figure 4:**
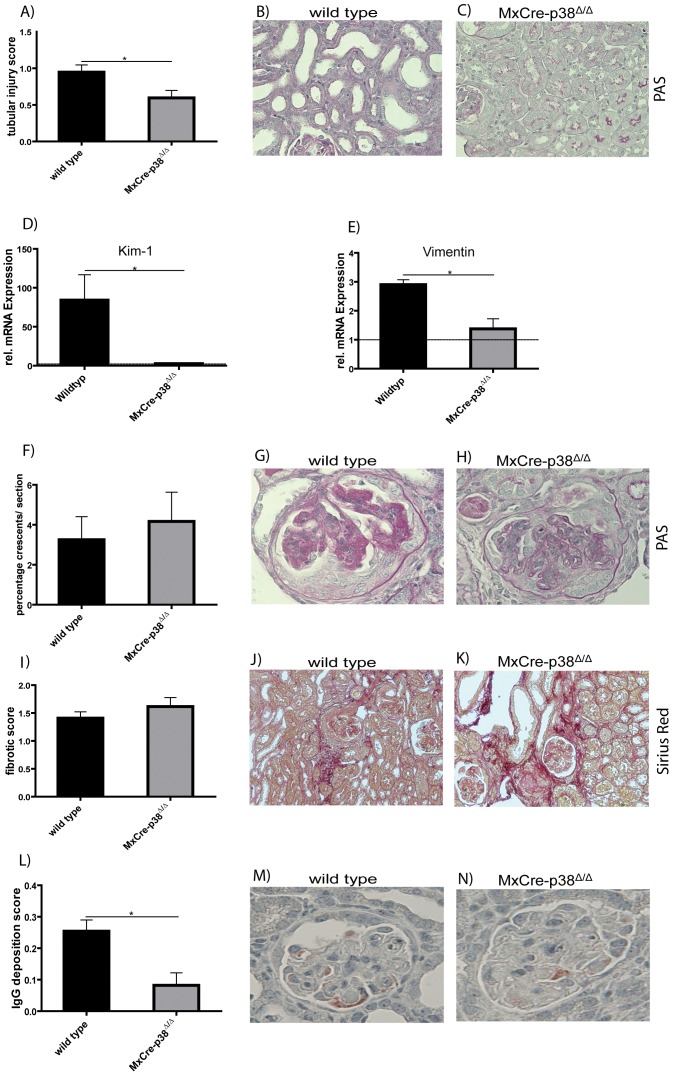
Deletion of p38α ameliorates tubular but not glomerular damage during anti-GBM nephritis. (A) Semi-quantitative scoring of tubular damage. p38α deletion significantly diminishes tubular damage (mean score 0.6±0.1, p<0.05 vs. control; n = 7) as compared to wild type mice (mean score 0.9±0.01; n = 7). Tubules of wild type mice (B) are dilated, whereas tubules of *MxCre-p38α^Δ/Δ^* (C) mice are still tightly packed. (D) KIM-1 mRNA level is dramatically increased in wild type mice (wild type 84.23±32.57; n = 4 vs. *p38α^Δ/Δ^* 2.675±1.248; n = 4) indicating high tubular damage. (E) Vimentin shows clear upregulation of its mRNA in wild type mice (2.900±0.1732; n = 4) whereas it is reduced nearly to the baseline level in *MxCre-p38α^Δ/Δ^* mice (1.375±0.3497; n = 4). Dashed line indicates RNA base level of control mice. (F–H) Analysis of crescent formation during anti GBM nephritis. Similar amounts of glomeruli revealed crescent formation in wild type (n = 6) and *MxCre-p38α^Δ/Δ^* mice (n = 6) (wild type: mean 3.3±1.2% vs. *MxCre-p38α^Δ/Δ^*: 4.2±1.5, p = ns). (I–K) Fibrotic tissue remodelling occurred in both wild type (n = 7) and *MxCre-p38α^Δ/Δ^* mice (n = 7) (wild-type: mean 1.409±0.1132 vs *MxCre-p38α^Δ/Δ^*: 1.615±0.1617, p = ns). Sirius red staining was performed 14 days after induction of anti-GBM nephritis. (L–M) p38α deletion affects the immune response to sheep IgG by decreased murine IgG depositions in the glomeruli.

To determine the effects of the absence of p38α on structural damage during anti- GBM nephritis, we performed qualitative and quantitative histological scoring. We first analyzed tubular damage using a semi-quantitative score (Fig. 4A–C). Interestingly, p38α deletion significantly diminished tubular damage (mean score 0.6±0.1, p<0.05) as compared to wildtype mice (mean score 0.9±0.1). KIM-1 and Vimentin mRNA levels corroborated these data, as these are also markers of tubular damage (Fig. 4D–E). In both cases mRNA levels in the knockout mice were significantly reduced in contrast to wild type mice (KIM-1: wildtype 84.23±32.57; n = 4 vs. *p38α^Δ/Δ^* 2.675±1.248; n = 4; Vimentin: wildtype 2.900±0.1732; n = 4 vs. *p38α^Δ/Δ^* 1.375±0.3497; n = 4). Next, we analyzed glomerular crescent formation semi-quantitatively (Fig. 4F–H), revealing that similar amounts of glomeruli revealed crescent formation in wild type and *MxCre-p38α^Δ/Δ^* mice (wild-type: mean 3.3±1.2% vs. *MxCre-p38α^Δ/Δ^*: 4.2±1.5, p = ns). Likewise, no difference was observed for the fibrotic score (Fig. 4I–K). A slight tissue remodelling occurred in both wildtype and *MxCre-p38α^Δ/Δ^* mice (wild-type: mean 1.4±0.1 vs *MxCre-p38α^Δ/Δ^*: 1.6±0.2, p = ns). Although the glomerular deposition of mouse IgG is generally very low there is a remarkably decrease in the *p38α^Δ/Δ^* group (0.082±0.0396; n = 5) in contrast to wildtype mice (0.25±0.035; n = 5; Fig. 4L–N). Thus, deletion of p38α protects from tubular damage in murine anti-GBM nephritis, whereas glomerular crescent formation and fibrosis is not affected.

### Recruitment of Leukocytes to Kidneys in Anti-GBM Nephritis is p38α-dependent

**Figure 5:**
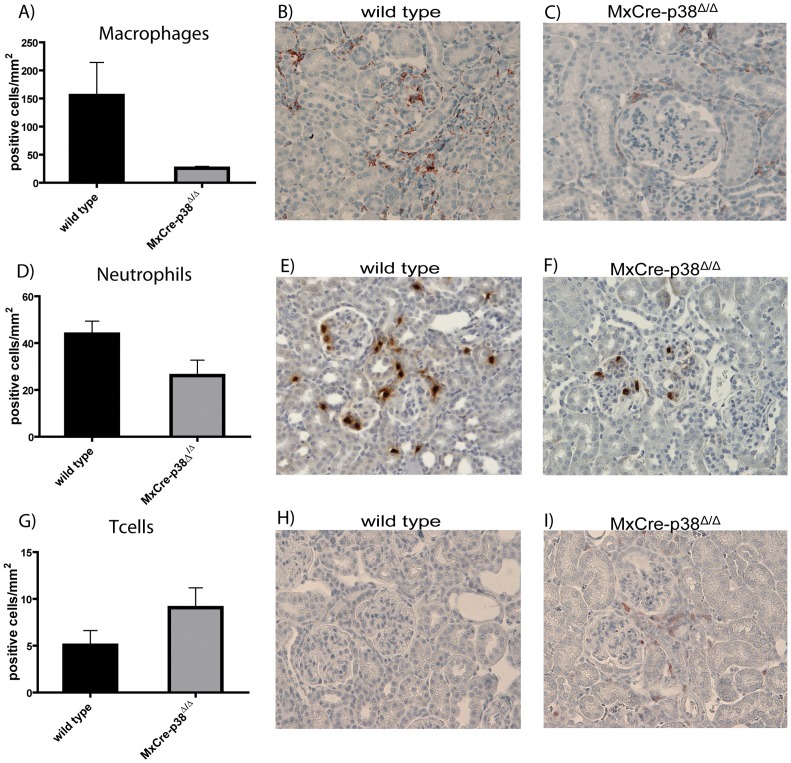
Recruitment of leukocytes to kidneys in anti-GBM nephritis is p38α-dependent. (A–C) F4/80 staining to detect macrophages clearly reveals a prominent infiltration in the kidneys of wild-type mice (mean 155.4±58.6 cells/mm^2^), whereas *MxCre-p38α^Δ/Δ^* mice show dramatically reduced macrophage numbers (mean 25.7±3.3 cells/mm^2^, p<0.05 vs. wild type). (D–F) Wild type mice show prominent neutrophil infiltration (mean 43.8±5.5 cells/mm^2^). There is a similar infiltration but less severe in *MxCre-p38α^Δ/Δ^* mice (mean 26.2±6.6 cells/mm^2^, p<0.05 vs. wild type). (G–I) In contrast to the other lymphocytes the number of T cells is not different among wild type (mean 5.0±1.5 cells/mm^2^) and *MxCre-p38α^Δ/Δ^* mice (mean 9.1±2.1 cells/mm^2^, p = ns vs. wild-type). Staining was performed 14 days after induction of anti-GBM nephritis (n = 7/group).

We next analyzed the role of p38α in leukocyte recruitment into the kidneys affected by anti-GBM nephritis. We thus performed immune phenotyping of leukocytes in kidney sections using immunohistochemistry and quantitatively scored leukocyte infiltration. We first asked whether macrophage infiltration is p38α-dependent. F4/80 staining to detect macrophages clearly revealed a prominent infiltration in the kidneys of wildtype mice (mean 155.4±58.6 cells/mm^2^). In contrast, *MxCre-p38α^Δ/Δ^* mice showed dramatically lower macrophage numbers (mean 25.7±3.3 cells/mm^2^, p<0.05 vs. wild type; Fig. 5A–C). A similar, although less strong difference was observed, when we evaluated the invasion of neutrophils. Whereas wild-type mice showed prominent neutrophil infiltration (mean 43.8±5.5 cells/mm^2^), this was less severe in *MxCre-p38α^Δ/Δ^* mice (mean 26.2±6.6 cells/mm^2^, p<0.05 vs. wild type; Fig. 5D–F). We then stained renal sections for B and T cells with anti-CD19 and anti-CD3 antibodies, respectively. Whereas we could not detect relevant B cell numbers in renal tissues, we clearly found T cell infiltration in wildtype mice injected with anti-GBM antibodies (mean 5.0±1.5 cells/mm^2^). In contrast to macrophages and neutrophils, the number of T cells was not different among wild type and *MxCre-p38α^Δ/Δ^* mice (mean 9.1±2.1 cells/mm^2^, p = ns vs. wildtype; Fig. 5G–I). These data indicate that p38α is critical for full activation of the innate immune response during anti-GBM nephritis.

### Inflammatory Gene Expression during Anti-GBM Nephritis is Partially p38α-dependent

**Figure 6:**
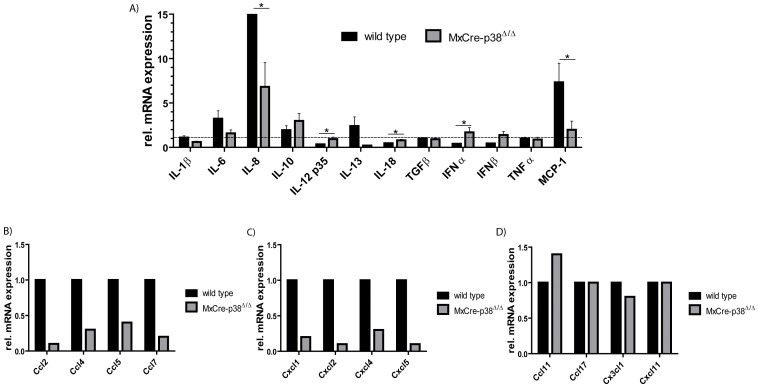
Inflammatory gene expression during anti-GBM nephritis is partially p38α dependent. qPCRs from renal tissue of wild-type and *MxCre-p38α^Δ/Δ^* mice 14 days after injection with anti-GBM antibodies. There is no difference in the mRNA expression of pro-inflammatory cytokines like TNF, IL-1β and IL-6. Furthermore there is a significant regulation of IL-8, IL-12 and IL-18. There are no significant changes in anti-inflammatory cytokines like IL-10, IL-13 and TGFβ1. MCP-1 is down-regulated in *MxCre-p38α^Δ/Δ^* mice. n = 4/group measured in duplicates (A). **Leukocyte infiltration is regulated by corresponding chemokines:** Chemokines participating in (B) macrophage and (C) neutrophil recruitment are remarkably downregulated in *MxCre-p38α^Δ/Δ^* mice. (D) Chemotactic regulation of T cells is unaffected in *MxCre-p38α^Δ/Δ^* mice. In each group the RNA of five mice was pooled and then analyzed by RT-PCR with a RT^2^ Profiler™ PCR Array kit. mRNA expression was normalized to beta-actin of wildtype mice.

As we have observed a reduced renal influx of macrophages and neutrophils, we next determined whether the altered recruitment of leukocytes is associated with a change in the expression of pro- and anti-inflammatory cytokines in the affected kidneys. We therefore performed qPCR from renal tissue of wildtype and *MxCre-p38α^Δ/Δ^* mice 14 days after injection with anti-GBM antibodies. Whereas the mRNA expression of the pro-inflammatory cytokines TNF, IL-1β and IL-6 was unaltered by p38α-deletion, we detected a significant downregulation of IL-8 as well as up- regulation of IL-12 and IL-18 in p38- deficient mice. In contrast, the expression of anti-inflammatory cytokines such as IL-10, IL-13 and TGFβ1 was not affected by p38α-deletion (Fig. 6A). In accordance with the data of macrophage staining, MCP-1 (monocyte chemoattractant protein 1) is clearly downregulated in *MxCre-p38α^Δ/Δ^* mice.

Because of the prominent phenotype of *MxCre-p38α^Δ/Δ^* mice revealing less macrophage and neutrophil influx as compared to wildtype mice, we analyzed mRNA expression of chemokines in more detail. As shown in Fig. 6B-C, chemokines associated with macrophage attraction were generally strongly down- regulated. Especially Ccl2, -4, -5 and -8 were massively suppressed in *MxCre-p38α^Δ/Δ^* mice. We also found that chemokines attracting neutrophils such as Cxcl1, -4, -5 and IL-8 were down regulated by p38α during anti-GBM nephritis. However, we could not detect such general changes regarding chemokines associated with lymphocyte influx (Fig. 6D). Taken together these data suggest that p38α deletion selectively blocks the expression of certain chemokines during anti-GBM nephritis, which are important for mononuclear cell influx to inflammatory sites. Despite these findings, pro-inflammatory cytokine production is not dramatically altered in these mice.

## Discussion

The p38MAPK family consists of four isoforms (p38α, p38β, p38γ and p38δ) and it has so far been largely unknown, which of these isoform is expressed and activated in renal inflammation. Our results indicate that the α-isoform is the most important one since we demonstrated only significant p38α activation during anti-GBM induced nephritis *in vivo*, while the other three p38 isoforms were not activated. In this study, we demonstrate accordingly a specific role of p38α in an experimental model of anti-GBM nephritis. Importantly, we demonstrate a preferential activation of p38α but not other p38 isoforms during the course of anti-GBM nephritis. Conditional deletion of p38α partially inhibited renal damage associated with anti-GBM nephritis by blocking leukocyte influx and tubular damage, while glomerular crescent formation and renal fibrosis were not affected.

Several lines of evidence suggested an important role for the p38MAPK pathway in the pathogenesis of crescentic GN: (i) p38MAPK is expressed and activated in an established rat model of crescentic GN [35]. These data have been further supported by expression studies in human biopsies containing various forms of crescentic GN including ANCA-associated GN [36]. Recent data also proved the expression and activation of downstream kinase MK2 in ANCA-associated GN further suggesting a functional pathway *in vivo* [36]. (ii) Phosphorylation and thus activation of p38MAPK occurs at sites of renal damage. Thus, immunohistochemical studies revealed active p38MAPK in podocytes, glomerular crescents, interstitial mononuclear infiltrates and tubular epithelial cells. In addition, the localization and extent of p38MAPK activation correlates with renal injury [37]. (iii) p38MAPK inhibitors have been successfully used in a rat crescentic GN model [27].

Inhibition of p38MAPKα and β with a small molecule inhibitor in a rat model of anti-GBM induced nephritis ameliorated both acute renal failure, proteinuria and leukocyte influx [38]. Sheryanna and colleagues confirmed these results showing, that p38MAPKα/β inhibition results in reduced renal expression of adhesion molecules and chemokines [27]. A concern about the inhibitors used in these studies is, however, their specificity. The applied compounds usually block both the α - and the β -isoform of p38MAPK and sometimes also affect non-p38MAPK, such as JNK3 [39].

Interestingly, overall survival and glomerular crescent formation were not significantly reduced in the absence of p38α. This is surprising, because p38MAPK is considered as crucial for acute inflammatory responses and, for instance, immune responses to LPS are highly dependent on intact p38MAPK signalling [40]. One explanation could be that other signalling pathways may substitute for p38α. This hypothesis is supported by the clinical data from p38MAPK inhibition in rheumatoid arthritis (RA) patients [28]. RA patients did show a transient decrease of inflammatory parameters but relapsed soon after the initiation of p38 inhibitor therapy. It was also shown that p38α deficiency could lead to increased NF-kB activation upon pro-inflammatory stimulation, which may rescue some of the effects elicited by p38 signalling [25].

We did not study the effect of p38α inhibition in other cell types involved in glomerular injuries such as mesangial and endothelial cells in vitro. In the latter, deletion of p38α may cause other effects in inflammatory pathways than in podocytes. Moreover, podocytes in vivo may react different to inflammatory stimuli in terms of p38 isoform activation than in an artificial in vitro culture system, which should be addressed in future studies. Alternatively, p38α may not be of major importance for glomerular inflammation and scarring. Indeed, recent evidence proves against a major role of p38 in the pathogenesis of rodent ANCA- mediated glomerulonephritis [41].

An explanation for the selective phenotype induced by p38α-deletion in anti-GBM induced glomerulonephritis emerges when looking more closely at the histological changes in the kidneys and cytokine expression profiling. Whereas glomerular crescent formation and interstitial scarring were not affected by p38α-deletion, suggesting that the fibrotic response in the anti-GBM GN model is not dependent on p38α, a significant amelioration of tubular damage was observed by us. The latter is in agreement with previous studies using p38 inhibitors [27]. Reduced tubular damage was associated with reduced leukocyte migration into the kidneys. Especially macrophage and neutrophil infiltration was significantly reduced, while T cell infiltration was comparable to wild type mice injected with anti-GBM antibodies.

Interestingly, p38α is the dominant p38MAPK isoform expressed and activated in macrophages and podocytes, which are regarded as crucially involved in the pathogenesis of crescentic glomerulonephritis. Moreover, we could not detect any reduction of renal expression of several pro- and anti-inflammatory cytokines such as TNF, IL-1, IL-6, IL-10 and TGFβ1. In contrast, we could even detect increases in renal mRNA expression of IL-12 and IL-18 indicating that p38α over time may also have a regulatory activity to limit inflammation in addition to its well-known pro-inflammatory role.

The reduced macrophage and neutrophil immigration into inflamed kidney tissue could be due to different chemokine expression. In fact, while chemokines responsible for lymphocyte influx were not generally altered, we could detect massive down-regulation of macrophage and neutrophil- attracting chemokines such as Ccl2, -4, Cxcl1, -4 and others. The latter finding may therefore explain the reduced influx of neutrophils and cells of the monocyte/macrophages into the kidneys during anti-GBM induced nephritis. However, these potentially positive effects did not translate into a major clinical amelioration of the experimental model.

In conclusion, p38α deletion selectively reduces inflammatory cell influx and tubular damage in murine anti-GBM nephritis but does not affect the formation of glomerular crescents and renal fibrosis. Consequently, full clinical and histological protection could not be achieved. Inhibition of p38α may therefore not be a promising strategy for the treatment for crescentic GN. Future studies could investigate why crescent formation and renal fibrosis can occur when macrophage infiltration is virtually absent.

## Supporting Information

Figure S1 **Pro-inflammatory cytokine stimulation specifically activates p38α in podocytes** ***in vitro*** **.** Analyses of the mRNA expression of the four 38MAPK isoforms and up- and downstream kinases in proliferating (A) and differentiated (B) podocytes reveal expression of the p38 α and γ isoform as well as of MKK3, MKK6, MK2 and ATF2. (C) Western blot analyses of the four p38MAPK isoforms support the findings of qPCR. (D) Protein was extracted from TNF-stimulated (10 ng/ml) differentiated podocytes and analysed for p38MAPK pathway activation using phospho-specific antibodies. (E) Immunoprecipitations (IP) were performed with buffer only (−) or with kidney lysates and anti-phospho p38MAPK antibody, or with kidney lysates using an isotype-matched control antibody (Iso). IPs and positive control lysate (+) were separated by SDS-PAGE, blotted onto nitrocellulose and probed with specific antibodies against p38 isoforms (arrows). HC: heavy chain of the precipitating antibody.
(TIFF)

Text S1 **Primer for genotyping and quantitative real-time PCR.**
(DOC)
