# Supplementary figures and images for: The Mitogen-Activated Protein Kinase p38α Regulates Tubular Damage in Murine Anti-Glomerular Basement Membrane Nephritis
Source: PLoS One. 2013 Feb 18;8(2):e56316. doi: 10.1371/journal.pone.0056316 (PMC3575386; doi:10.1371/journal.pone.0056316)

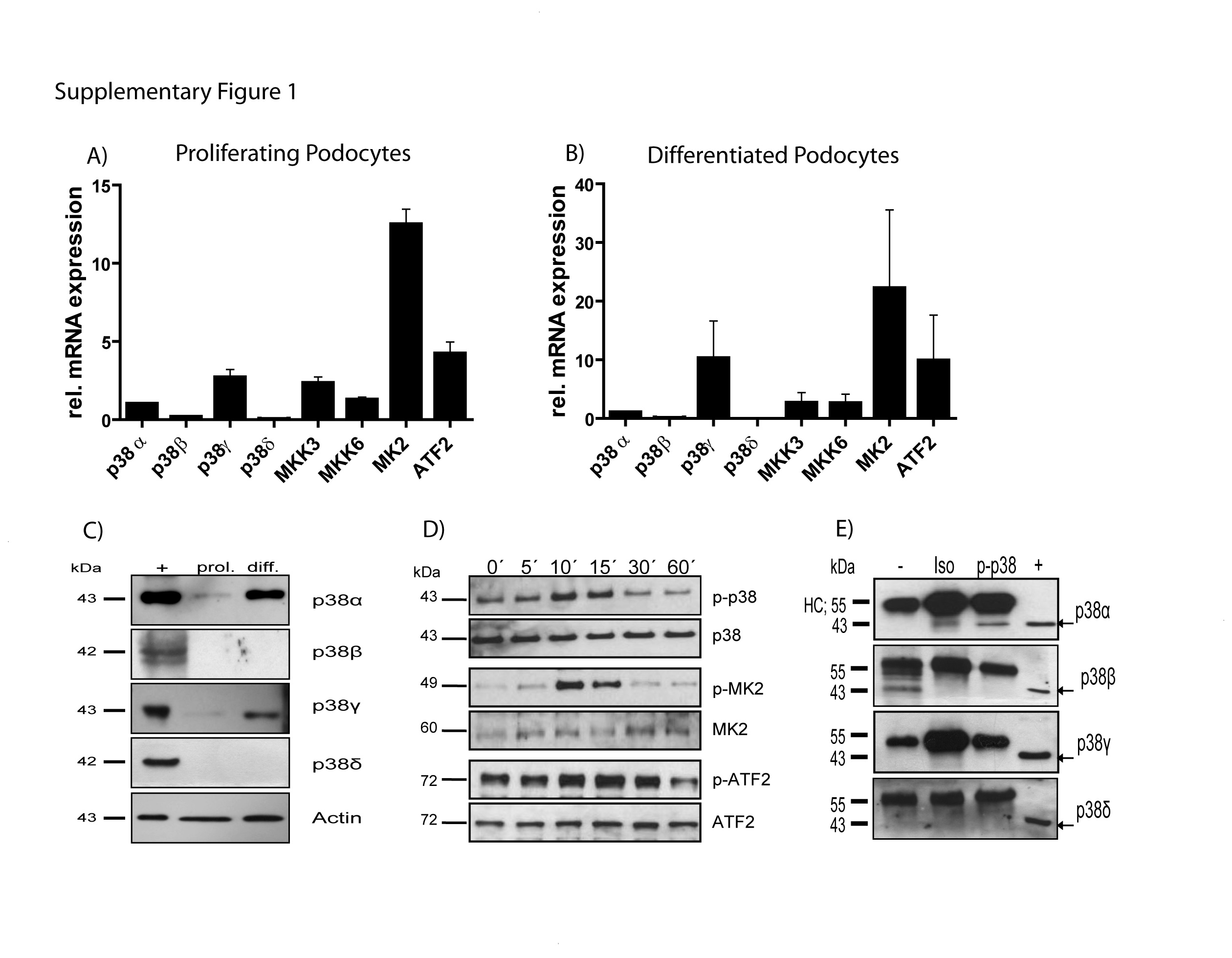

Supplement: Figure S1 — Pro-inflammatory cytokine stimulation specifically activates p38α in podocytes in vitro . Analyses of the mRNA expression of the four 38MAPK isoforms and up- and downstream kinases in proliferating (A) and differentiated (B) podocytes reveal expression of the p38 α and γ isoform as well as of MKK3, MKK6, MK2 and ATF2. (C) Western blot analyses of the four p38MAPK isoforms support the findings of qPCR. (D) Protein was extracted from TNF-stimulated (10 ng/ml) differentiated podocytes and analysed for p38MAPK pathway activation using phospho-specific antibodies. (E) Immunoprecipitations (IP) were performed with buffer only (−) or with kidney lysates and anti-phospho p38MAPK antibody, or with kidney lysates using an isotype-matched control antibody (Iso). IPs and positive control lysate (+) were separated by SDS-PAGE, blotted onto nitrocellulose and probed with specific antibodies against p38 isoforms (arrows). HC: heavy chain of the precipitating antibody. (TIFF) [file pone.0056316.s001.tif]
