# Supplementary material for: The Mitogen-Activated Protein Kinase p38α Regulates Tubular Damage in Murine Anti-Glomerular Basement Membrane Nephritis
Source: PLoS One. 2013 Feb 18;8(2):e56316. doi: 10.1371/journal.pone.0056316 (PMC3575386; doi:10.1371/journal.pone.0056316)
Supplement: Text S1 — Primer for genotyping and quantitative real-time PCR. (DOC) [file pone.0056316.s002.doc]

Genotyping of mice was performed using the following primers: p38α (320 bp) 5´-GCAGGGTAGAGAAGCATCCCA-3´and 5´-GAACGATACCAGAACCTGTCC-3´, cre (600 bp) 5´-CGGTCGATGCAACGAGTGATGAGG-3´and 5´-CCAGAGACGGAAATCCATCGCTCG-3´, floxed (500 bp) 5´-GCAGGGTAGAGAAGCATCCCA-3´ and 5´-GGATGTGGAATGTGTGCGAGG-3´.

Murine primers for quantitative real-time PCR were as follows: p38α agctgtcgagaccgtttcag and agcttctggcacttcacgat, p38β atgtagcggtgaacgaggac and acctccatgattcgcttcag, p38γ ggaaggcctccctgagttag and agggtgcggtctacatcatc, p38δ cgccacacagacactgagat and atgatgcaaccaacagacca, MKK3 cacggtcgactgcttctaca and agcttgctatgcaggtgctc, Mkk6 gtccattcaccgtgaccttc and ggcccagtgtgttaatgagc, MAPKAPK-2 AACGATGGGAGGATGTCAAG and AGGGTTGGATGCGTCTTCTA, ATF2 gactccaacgccaacaagat and tgaggtaaagggctgtcctg, Actin tgtccaccttccagcagatgt and agctcagtaacagtccgcctaga, tnfα Gctgagctcaaaccctggta and cggactccgcaaagtctaag, TGFβ1 tggagcaacatgtggaactc and Agacagccactcaggcgtat, IL1α acgtcaagcaacgggaag and tgctgatctgggttggatg IL1β CAGGCAGGCAGTATCACTCA and AGGTGCTCATGTCCTCATCC, IL8 AGCACCAGCCAACTCTCACT and CGTTAACTGCATCTGGCTGA, IL10 gccttatcggaaatgatcca and atcctgagggtcttcagcttc.
